# French Intensive Care Society, International congress – Réanimation 2016

**DOI:** 10.1186/s13613-016-0114-z

**Published:** 2016-06-17

## **Correspondence:** Boris Jung - boris.jung@me.com

*Annals of Intensive Care* 2016, **6(Suppl 1)**:O5

**Introduction** Although rapid response systems (RRSs) are known to reduce in hospital cardiac arrest rate, their effect on mortality remains in question. The present study aimed at evaluating the effect of a medical emergency team (MET) implementation on mortality in hospitalized patients.

**Patients and methods** A prospective study was conducted in the four hospitals of the regional healthcare center of Montpellier, France. An intensivist-led MET was implemented on a 24/7 basis in only one of the four hospitals from January 2012 to June 2012. Patients >18 years admitted for more than 24 h in the medical-surgical wards from July 2010 to December 2011 (pre-MET period) and from July 2012 to December 2013 (MET period) were included. The main outcome was unexpected mortality in hospitalized patients. An updated systematic review and meta-analysis were also performed.

**Results** A total of 137,251 patients were admitted for 24 h or more in the medical-surgical wards during the pre-MET and MET periods. MET implementation was associated with a decrease in unexpected mortality rate in the hospital that implemented MET (from 21.9 to 17.4 per 1000 admissions; *P* = 0.002). Reduction in unexpected mortality associated with MET implementation could be estimated at 1.5 lives saved per week in the MET hospital. In the three other hospitals, mortality rate was not significantly modified (from 19.5 to 19.9 per 1000 admissions; *P* = 0.69). Patients in the MET hospital were more frequently admitted to the intensive care unit (ICU) during the MET period (45.8 vs 52.9 per 1000; *P* = 0.002), and their sequential organ failure assessment (SOFA) score upon ICU admission significantly decreased from 7 [4–10] to 5 [2–9]; *P* < 0.001. The updated meta-analysis including the present results showed that RRS was associated with a significant decrease in both unexpected (OR 0.51; 95 % CI 0.35–0.76) and overall mortality (OR 0.89; 95 % CI 0.85–0.93).

**Fig. 1:**
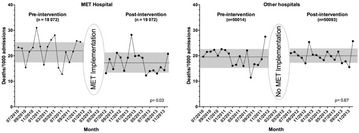
Evolution of unexpected death rate per 1000 admissions by month in the MET pavilion (*left side*) and in the three other pavilions (*right side*). *Dotted lines* represent the mean rate per month. *Grey rectangles* represent the standard deviations

**Conclusion** In the present prospective study, implementation of a MET was associated with a decrease in unexpected and overall mortality. Updated meta-analysis confirms the benefit of RRS on unexpected and overall mortality (Fig. 1).

**Competing interests** None.

