# Supplementary material for: French Intensive Care Society, International congress – Réanimation 2016
Source: Ann Intensive Care. 2016 Jun 17;6(Suppl 1):1–236. doi: 10.1186/s13613-016-0114-z (PMC4912513; doi:10.1186/s13613-016-0114-z)
Supplement: Related texts

# Reply: P167 Proteomic response and adaptation mechanisms of E. coli 536 to overcome the inhibitory effect of cranberry proanthocyanidins

**Authors:** Dimitri Margetis, Odile Bouvet, Didier Chevret, Didier Dreyfuss, Erick Denamur, Jean-Damien Ricard

**Affiliations:** 954 0000 0001 2150 9058 grid.411439.a Anesthésie réanimation, Pitié-Salpêtrière Hospital, Paris, France; 955 0000 0001 2217 0017 grid.7452.4 Iame - inserm umr1137, Université Paris Diderot, Paris, France; 956 grid.417961.c 78, I.N.R.A (Institut National de Recherche Agronomique), Jouy-en-Josas, France; 957 0000 0001 2217 0017 grid.7452.4 Inserm, iame, umr 1137, Université Paris Diderot, Sorbonne Paris Cité, Paris, France; 958 0000 0001 2217 0017 grid.7452.4 Iame inserm umr1137, Université Paris Diderot, Paris, France; 959 Service de Réanimation Médico-Chirurgicale, CHU Louis Mourier, Colombes, France

## **Correspondence:** Dimitri Margetis - dimargetis@free.fr

*Annals of Intensive Care* 2016, **6(Suppl 1)**:P167

**Introduction** The cranberry fruit (*Vaccinium macrocarpon*) is particularly rich in polyphenols (tannins), and among these, the primary active compound is the A-type proanthocyanidin (cPAC), which exhibit potent antibacterial properties. In a previous study, we have made evidence of the strong inhibitory effect of cPAC on growth, adhesion and virulence of oropharyngeal and lung isolates of *E. coli* [1].

The present study was undertaken to establish a baseline of knowledge of the molecular responses of the highly virulent *E. coli* strain *536* during growth in the presence of cPAC. We employed proteomic analysis and made complementary experiences to characterize the global response of exponential and stationary phases *E. coli* 536 grown in a nutrient-rich broth medium supplemented with 1 mg/mL, cPAC as compared with a reference culture grown without cPAC.

**Materials and methods***E. coli* 536 exposed or not to 1 mg/ml of cPAC proteome was obtained in exponential and stationary growth phase by mass spectrometry proteomic analysis LC–MS/MS.

Evaluation of the intracellular ROS level by the dichlorofluorescein diacetate DCFH-DA method was evaluated using a spectrofluorimeter. Membrane potential, cell size and cell viability were evaluated using a flow cytometer (Guava EasyCyte Plus, Millipore). To examine the changes of membrane potential after cPAC exposure, the cells were stained with DiOC6(3) (3,3′-dihexyloxacarbocyanine iodide) and then immediately analyzed using a flow cytometer. The fluorescence-based LIVE/DEAD kit was used to examine the cell viability.

**Results** A total of 641 proteins were detected, and among these, 483 were in both exponential and stationary growth phases, whereas 506 and 618 proteins were detected only during the exponential and stationary growth phases, respectively. Proteins were manually grouped into 25 metabolic pathway categories. Among the 641 identified proteins, 117 had a significant effect, and among these, 11 were in both exponential and stationary growth phases, whereas 68 and 60 proteins were detected only during the exponential and stationary growth phases, respectively. Considerable differences were also observed in protein abundances between the untreated and treated cells. During the exponential and stationary growth phases, many proteins were over-abundant (31 and 29, respectively) and under-abundant (37 and 31, respectively).

The characterization of 117 proteins with a significant effect revealed that more proteins were over-abundant in different functional categories, especially those involved in glycolyse, fermentation, iron metabolism and detoxification. In contrast, the under-abundant proteins were mainly implicated in transport, TCA cycle and respiration.

cPAC exposure mainly affected the pathways of iron metabolism with strong evidence of iron privation, cellular detoxification as response to the oxidative stress exerted by cPAC and a redirection of respiratory metabolism through the fermentative *pathways.* cPAC treatment also alters many membrane functions by the strong inhibition of membrane proteins transport and secretion, affecting the respiratory chains, fimbrial expression, cell division and proton-motrice force.

Supplementary experiments showed no effect of cPAC on bacterial viability, a size increase as result of cellular division inhibition and a drastic reduction of the proton-motrice force.

ROS dosage showed a significant decrease during stationary phase of growth in cPAC treated bacteria as response to the oxidative stress exerted by proanthocyanidins.

**Conclusion** Proteomic analysis indicates the metabolic pathways affected by cPAC and *E. coli* adaptation. Treated bacteria are in a state of iron limitation, energy metabolism is redirected to fermentation and membrane proteins secretion and transport systems strongly inhibited. As a result, we observe a fimbriation inhibition affecting bacterial mobility, adhesion and virulence and alteration of the proton-motrice force and cellular division in *E. coli*. Our results highlight strategy adapted by *E. coli* 536 to resist the toxicity of tannins. During exposure to cranberry, *E. coli* is subjected to complex metabolic adaptations that aim to reduce the rate of intracellular ROS. The lack of production of fimbriae caused by cranberry contributes to the permanent activation of the oxidative stress response.

**Competing interests** None.

Reference

1. Margetis, D., D. Roux, et al. (2015). “Effects of Proanthocyanidins on Adhesion, Growth, and Virulence of Highly Virulent Extraintestinal Pathogenic *Escherichia coli* Argue for Its Use to Treat Oropharyngeal Colonization and Prevent Ventilator-Associated Pneumonia.” Crit Care Med 43(6): e170–8.

# Reply: P204 Modulation of inflammatory response related to severe peritonitis by polymyxin-B hemoperfusion

**Authors:** Remi Coudroy, Jean-Claude Lecron, Didier Payen de la Garanderie, René Robert, ABDOMIX Group

**Affiliations:** 1080 0000 0000 9336 4276 grid.411162.1 Réanimation médicale, CHU de Poitiers, Poitiers, France; 1081 0000 0000 9336 4276 grid.411162.1 Litec ea4331, Chu De Poitiers, Poitiers, France; 1082 0000 0000 9725 279X grid.411296.9 Réanimation chirurgicale, CHU Lariboisière, Paris, France

## **Correspondence:** Remi Coudroy - remi.coudroy@chu-poitiers.fr

*Annals of Intensive Care* 2016, **6(Suppl 1)**:P204

**Introduction** Although polymyxin-B hemoperfusion is supposed to improve outcomes in patients with sepsis by adsorbing endotoxin and decreasing systemic inflammation, the recent ABDOMIX study did not demonstrate any beneficial effect of polymyxin-B hemoperfusion in patients with septic shock due to peritonitis. In this context, cytokine clearance induced by polymyxin-B hemoperfusion is debated. The objectives of the study were to assess the influence of polymyxin-B hemoperfusion on plasma cytokine concentrations and to identify the cytokines associated with day 28 mortality.

**Patients and methods** This ancillary study of the ABDOMIX study investigated the impact of two polymyxin-B hemoperfusion sessions on day 28 mortality in peritonitis-induced septic shock. Blood samples were taken within 10 h post-surgery (P1), at the end of the first hemoperfusion session in the experimental group or 2 h after P1 in the control group (P2), 24 h after P1 (P3), and at the end of the second hemoperfusion session in the experimental group or 2 h after P3 in the control group (P4). Cytokines such as tumor necrosis factor α, interferon γ, interleukin-1β, interleukin-1α, interleukin-1RA, interleukin-6, interleukin-4, interleukin-10, interleukin-17A and interleukin-22 were assessed using magnetic bead-based immunology multiplex assay or enzyme-linked immunosorbent assay.

**Results** Among the 232 patients included in the ABDOMIX study, 19 patients were excluded due to missing sampling and 213 patients (109 in the hemoperfusion group and 104 in the control group) were consequently included in the analysis. Postoperative P1 cytokine concentrations were not different between the 2 groups. Cytokine variation between P1 and P2 was not different between the 2 groups except for interleukin-1RA and interleukin-10, which decreased more pronouncedly in the control group than in the hemoperfusion group (*p* = 0.016 and 0.047, respectively). Cytokine variations between P3 and P4 were not different between the 2 groups except for IL-10 and IL-22, both of which decreased in the control group, whereas both of them increased in the hemoperfusion group (*p* = 0.002 and 0.04, respectively). Using a logistic stepwise regression model, the absence of decrease in interleukin-10 and interleukin-22 between P1 and P3 was associated with day 28 mortality independently from Simplified Acute Physiology Score II (*p* = 0.002 and 0.04, respectively).

**Conclusion** Polymyxin-B hemoperfusion was not associated with a decrease in cytokine concentrations as compared to the control group. Higher levels of interleukin-10 and interleukin-22 in polymyxin-B hemoperfusion group may suggest unexpected deleterious interference between these cytokines and PMX membrane. The absence of decrease in interleukin-10 or interleukin-22 blood concentrations within 24 h after peritonitis-related septic shock was independently associated with day 28 mortality.

**Competing interests** None.

# Reply: S45 Impaired biological response to aspirin in comatose patients resuscitated from out-of-hospital cardiac arrest

**Authors:** Jean-François Llitjos, Ludovic Drouet, Sebastian Voicu, Nicolas Deye, Bruno Megarbane, Patrick Henry, Jean-Guillaume Dillinger

**Affiliations:** 1417 0000 0001 0274 3893 grid.411784.f Réanimation Médicale, Hôpital Cochin, Paris, France; 1418 0000 0000 9725 279X grid.411296.9 Department of Angio-Hematology, Hospital Lariboisière, Paris, France; 1419 0000 0000 9725 279X grid.411296.9 Intensive Care Unit, Hospital Lariboisière, Paris, France; 1420 0000 0000 9725 279X grid.411296.9 Réanimation Médicale et Toxicologique, Hôpital Lariboisière, Paris, France; 1421 0000 0000 9725 279X grid.411296.9 Service de Réanimation Médicale et Toxicologique, CHU Lariboisière, Paris, France; 1422 0000 0000 9725 279X grid.411296.9 Cardiology, Hospital Lariboisière, Paris, France

## **Correspondence:** Jean-François Llitjos - jllitjos@gmail.com

*Annals of Intensive Care* 2016, **6(Suppl 1)**:S45

**Introduction** Acute coronary syndromes are one of the main causes of out-of-hospital cardiac arrest. These patients are at high risk of persistent platelet reactivity under aspirin treatment. We herein evaluate the biological response to aspirin and ADP inhibitors in comatose patients resuscitated from out-of-hospital cardiac arrest treated by either oral or intravenous aspirin, with or without an ADP inhibitor.

**Patients and methods** Patients were randomized to receive long-term antiplatelet treatment with either 100 mg oral or 100 mg intravenous aspirin and when necessary dual antiplatelet therapy after emergency coronary angiography. Patient’s blood samples were obtained at day 3 and day 7 after cardiac arrest. Biological response to aspirin was assessed 24 h after last aspirin intake using light transmission aggregometry with arachidonic acid and the whole blood point-of-care assay PFA-100® system with collagen–epinephrin. Biological response to ADP inhibitors was assessed using flow cytometry (vasodilator-stimulated phosphoprotein platelet reactivity index), light transmission aggregometry with adenosine diphosphate and PFA-100® system with collagen–adenosine diphosphate.

**Results** Twenty-two consecutive patients were prospectively included. Thirteen patients received aspirin in the intravenous route and 9 patients in the oral route. Clinical, demographical, biological and angiographical characteristics were similar in both groups. Using light transmission aggregometry with arachidonic acid, 10 patients had persistent platelet reactivity, corresponding to 45 % of the total population. At day 3 after cardiac arrest, we found a significantly higher maximum intensity in the oral group when compared to the IV group (29 vs. 15 %, *p* = 0.04). The same way, closure time was significantly faster in the oral group when compared to the IV group (155 vs. 277 s, *p* = 0.04). We found no difference at day 7 after cardiac arrest. Irrespective of the biological test, clopidogrel was associated with a biological inefficacy at day 3. Under prasugrel, persistent platelet reactivity was found in 20 % of samples with inadequate effect using flow cytometry, in 10 % using light transmission aggregometry with adenosine diphosphate and in 40 % using the PFA-100® system with collagen–adenosine diphosphate. Stent thrombosis occurred in 1 patient treated with oral aspirin and efficiently by prasugrel. We found no difference in mortality in both groups.

**Conclusion** In this study, impaired response to aspirin both oral and intravenous is frequent in comatose patients resuscitated from out-of-hospital cardiac arrest, with a higher rate using the oral route. The use of prasugrel as an ADP inhibitor seems to be associated with a highest efficiency in inhibiting platelet reactivity than clopidogrel in this population of patients.

**Competing interests** None.

# Reply: S49 Ex vivo effects of hyperglycemia on phenotype and production of reactive oxygen species by the NADPH oxidase of human immune cells in acute inflammatory response

**Authors:** Benjamin Soyer, Valérie Faivre, Charles Damoisel, Anne-Claire Lukaszewicz, Didier Payen de la Garanderie

**Affiliations:** 0000 0000 9725 279X grid.411296.9 Réanimation chirurgicale, CHU Lariboisière, Paris, France

## **Correspondence:** Benjamin Soyer - benjaminsoyer@wanadoo.fr

*Annals of Intensive Care* 2016, **6(Suppl 1)**:S49

**Introduction** Glucose is the main energy substrate (1) for immune cells (monocytes (monos) and polymorphonuclears (PMNs)). Glucose has also a pro-inflammatory action through the production of reactive oxygen species (ROS).

Objectives: (1) to demonstrate an increase in ROS production during moderate hyperglycemia through the NADPH oxidase (NADPHox) pathway (NADPH comes from the pentose shunt pathway (PSP)); (2) to evaluate the phenotypic consequences on immune cells: monos, PMNs, and lymphocytes (lymphos).

**Materials and methods** Whole blood from healthy volunteers (CCPSL UNT-No13/SL/015) was incubated 90 min in: 1—normoglycemia (NG) or 2—hyperglycemia (HG, glucose 12.5 mM), or with deoxyglucose, a glycolysis blocker (DOG 12.5 mM), or with specific inhibitors of PSP: Epi Androsterone and 6 Aminonicotinamide (EPI 6AN).

Measurements: 1—overall ROS production with luminometry (2) technic (area under the curve (AUC) and slope of activation) at baseline and after stimulation with PMA-Ionomycin (Phorbol-12-Myristate-13-Acetate, Ionomycin). 2—Phenotypic changes in immune cells:

• Monos (CD14+): HLA-DR, CD11b, CD62L (markers of cellular activation)

• PMNs (CD16+): CD11b, CD62L

• T lymphos (CD3+): CTLA4 (marker of cellular inhibition)

• B lymphos (CD19+): HLA-DR

Statistics: nonparametric tests.

**Results** ROS production: at baseline, AUC (*n* = 19) in HG was higher than that in NG (*p* < 0.05) (increased production). After stimulation with PMA–ionomycin, this difference disappeared. Inhibitors reduced the AUC compared to the NG (*p* < 0.05). The slope of luminometry (reactivity) was higher in HG than in NG (*p* < 0.05). The slopes in NG and HG were higher than those observed with inhibitors in NG and HG (*p* < 0.05).

1. Phenotypic changes: no difference between monos, PMNs and lymphos in the 3-glycemic conditions (there were only few decreases for some markers in DOG conditions).

**Discussion** Hyperglycemia increased ROS production of immune cells in basal condition. However, in stimulated conditions, hyperglycemia did not increase ROS production of the NADPH oxidase system but accelerated the reactivity of the system (slope of the luminometry curve). Nonspecific inhibitor (DOG) and specific inhibitors of PSP (EPI 6AN) decreased ROS production and reactivity of NADPHox system.

**Conclusion** Our experiments confirm the implication of the PSP in ROS production. Functional changes induced by hyperglycemia were not associated with phenotypic alterations in immune cells.

**Competing interests** None.

References

1. Pearce, E. L. & Pearce, E. J. Metabolic pathways in immune cell activation and quiescence. Immunity 38, 633–643 (2013).

2. Lukaszewicz, A.-C., Gontier, G., Faivre, V., Ouanounou, I. & Payen, D. Elevated production of radical oxygen species by polymorphonuclear neutrophils in cerebrospinal fluid infection. Ann. Intensive Care 2, 10 (2012).
